# An exploration of the experience of dapagliflozin in clinical practice

**DOI:** 10.2144/fsoa-2022-0038

**Published:** 2022-11-01

**Authors:** Anuba Paulvarnan Anitha, Swetha Balasubramanian, Arun Gopal Ramalingam, Shini Rubina Samuel Kennady, Nila Ganamurali, Dhivya Dhanasekaran, Sarvesh Sabarathinam

**Affiliations:** 1 Department of Pharmacy Practice, SRM College of Pharmacy, SRM IST, Kattankulathur, Kancheepuram, Tamil Nadu, 603203, India; 2 Drug Testing Laboratory, Interdisciplinary Institute of Indian System of Medicine (IIISM), SRM Institute of Science & Technology, Kattankulathur, Tamil Nadu, 603203, India

**Keywords:** clinical pharmacist, clinical research, CYP3A4, dapagliflozin, diabetes, drug interaction, hyperglycemic, metabolism, pharmacokinetics, sodium-glucose cotransporters-2

## Abstract

Despite the availability of established treatments, heart failure (HF) is associated with a poor prognosis and suboptimal management, highlighting the need for new treatment and prevention options. It is suggested that sodium-glucose cotransporters 2 inhibitors can provide a beneficial therapeutic approach to significantly lower the disease burden associated with cardiovascular illness in both patients with and without Type 2 diabetes mellitus. This review focuses on the therapeutic aspects of dapagliflozin in clinical practice. Future studies may intend to confirm the significant clinical benefits of sodium-glucose cotransporter-2 inhibitors.

Despite the availability of established treatments, heart failure (HF) is associated with a poor prognosis and suboptimal management, highlighting the need for new treatment and prevention options. Cardiovascular complications are common in patients with Type 2 diabetes mellitus, significant with HF. As a result, several cardiovascular outcome trials have engaged in studies related to glucose lowering therapies and their impact on cardiovascular outcomes. Sodium-glucose cotransporters 2 (SGLT-2) inhibitors have shown beneficial effects on cardiovascular illness. SGLT-2 inhibitors are a novel class of oral antidiabetic agents indicated worldwide for the user management of Type 2 diabetes mellitus [1]. At present the therapeutic class comprises three agents, they are canagliflozin, dapagliflozin and empagliflozin [2]. SGLT-2 cotransporters are found in the kidney present at early proximal converted tubule(PCT) where the reabsorption of glucose occurs actively [3]. Inhibition of such cotransporter in the kidney halts the glucose reabsorption and increases the excretion of glucose via the urine, thereby reducing blood glucose level [4]. As this mechanism is independent of the action of insulin, they rarely cause hypoglycemia [5]. In addition to glucose control, inhibition of SGLT-2 cotransporters also promote a reduction in blood pressure and plasma uric acid levels and hence acting as cardioprotective and renoprotective. SGLT-2 inhibitors, targeting cardiometabolic abnormalities, potentially have cardioprotective action [6,7]. The mechanism of SGLT-2 inhibition is summarized in Figure 1 dapagliflozin is a potent and selective inhibitor of SGLT-2 with more advantages. It has demonstrated linear pharmacokinetics over the 2.5–500 mg/day dosing range, and is unaffected by ingesting meals, and is mostly excreted in the urine. The decreased incidence of hypoglycemia reported in the studies, which most likely happens because dapagliflozin operates independently of the glucose dependent production of insulin by the pancreatic beta cells, was another therapeutic benefit of dapagliflozin. Some studies that compared the effectiveness of dapagliflozin with empagliflozin revealed that dapagliflozin showed reduced incidence of HF when compared with other SGLT-2 inhibitors. Importantly, the main advantage of dapagliflozin over other SGLT-2 inhibitor is that dapagliflozin shown to improve the prognosis of HF with diminished systolic function without affecting blood sugar levels. A study that analyzed costs and benefit ratio for the use of dapagliflozin emphasized that dapagliflozin was more affordable, improved the quality-adjusted life-year and was more cost-effective when compared with other glucose lowering agents. As several studies proved the effectiveness of dapagliflozin in broad spectrum population when compared with other SGLT-2 inhibitors, in this context we aim to explore the application of dapagliflozin in clinical practice. In this viewpoint we discuss the clinical evaluation of the use of dapagliflozin in the treatment of cardiovascular illness.

**Figure 1.**
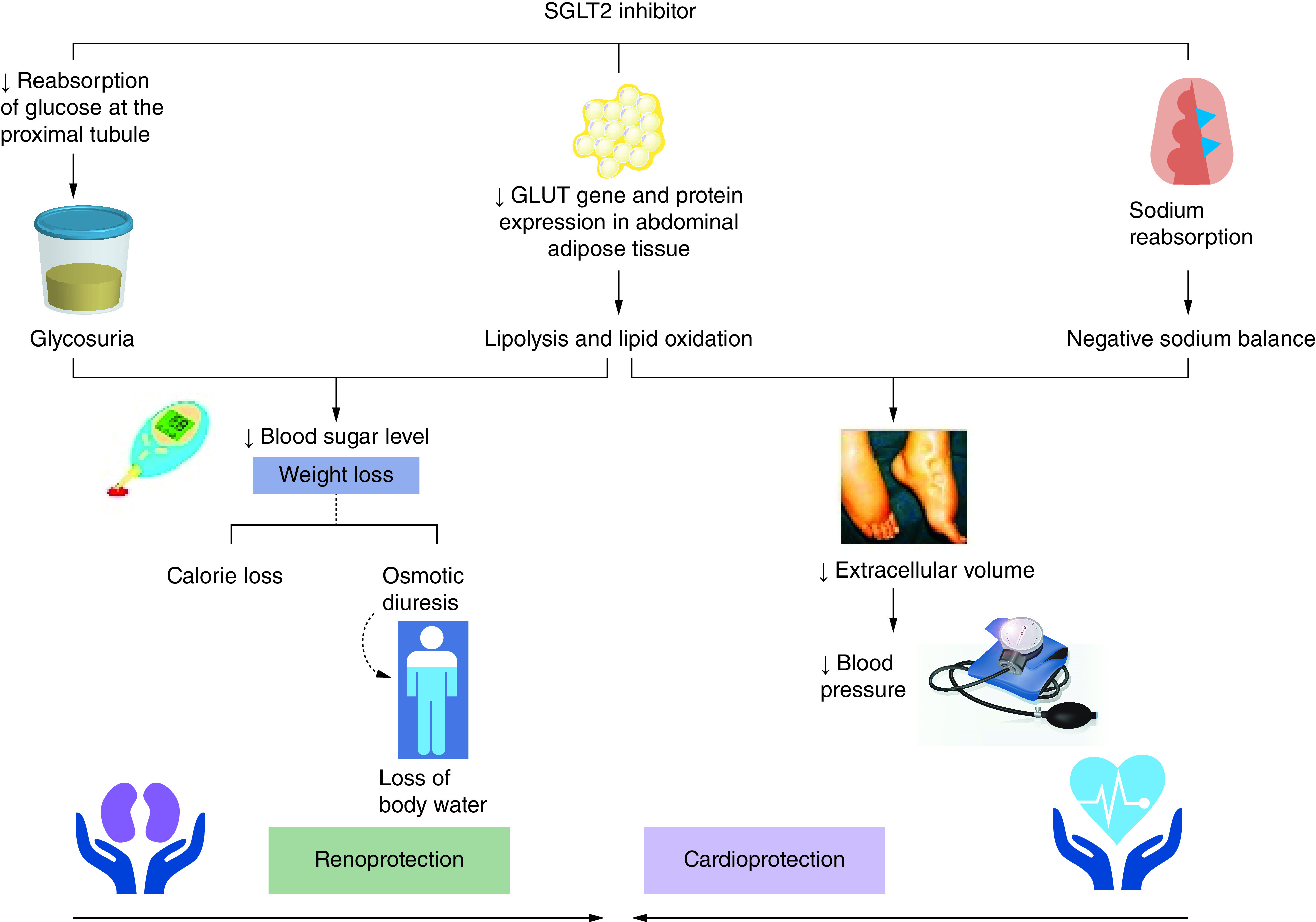
Action of SGLT-2 inhibitor.

## Practical questions

### Renoprotective action of dapagliflozin

The kidney absorbs approximately 90% of glucose via SGLT-2 cotransporter, which is found on the apical side of proximal tubule cells and transports sodium and glucose at the same time [8]. SGLT-2 proteins can be upregulated in Type 2 diabetes patients, increasing the renal threshold for glucose reabsorption resulting in worsening of glycemic control. Renal hyper filtration occurs as a result of increased glucose reabsorption resulting in acute renal damage. Limiting glucose transport in human proximal tubular cells may reduce the inflammatory response and prevent fibrosis of kidney tubules. SGLT-2 inhibitors are newly developed oral hypoglycemic agents that target the kidney and block glucose reabsorption to achieve glycemic control; thereby, providing a protective action on the kidney beyond glucose lowering effects [9].

### Cardioprotective action of dapagliflozin

SGLT-2 inhibitors, a new class of antidiabetic drugs, have been shown to have cardiac protective effects in addition to glucose lowering effects such as visceral fat reduction and inhibition of inflammation and oxidative stress. The mechanisms underlying these clinical benefits may not be fully explained by pathogenetic scenarios based on the effects of canonical SGLT-2 inhibitors. According to new evidence in cardio myocytes, SGLT-2 inhibitors can inhibit the Na^+^/H ^+^ (sodium–proton) exchanger, resulting in a decrease in intracellular sodium ions (Na^+)^ and calcium ions (Ca^2+^) concentrations with increasing mitochondrial Ca^2+^ concentrations, ultimately optimizing cardiac mitochondrial function and energetics [10]. This off-target effect of SGLT-2 inhibitors may illustrate, at least in part, the beneficial effects of SGLT-2 inhibitors treatment on HF.

### Poly pharmacotherapy: can dapagliflozin be used in the poly treated patient ?

Experts believe in the safety and tolerability profile of dapagliflozin. In patients with Type 1 diabetes, dapagliflozin as an adjunct therapy to insulin caused significant changes in HbA1C (glycated hemoglobin), fasting and postprandial glucose and atherogenic lipids [11]. A randomized, double-blind, placebo-controlled trial in patients with HF and reduced ejection fraction found that using or not using glucose lowering therapy similarly improved outcomes. Dapagliflozin may thus represent a novel therapeutic approach, though long-term controlled clinical trials with a larger patient population are required to confirm the currently available data [12].

### Can dapagliflozin be used in a patient with renal & hepatic impairment patient?

Dapagliflozin systemic exposure is higher in patients with severe renal or hepatic impairment. Dapagliflozin is primarily eliminated through metabolism (primarily glucuronidation via UDP-glucuronosyltransferase) [13]. The impact of renal impairment on dapagliflozin metabolism was manifested by decreased metabolic clearance of dapagliflozin. According to the literature, subjects with moderate and severe renal impairment have higher systemic exposure to dapagliflozin at a steady state than subjects with normal renal function. Dapagliflozin becomes largely ineffective as the estimated glomerular filtration rate (eGFR) approaches 45 ml/min/1.73 m^2^. As a result, the prescribing information for dapagliflozin recommends not starting it in patients with an eGFR less than 60 ml/min/1.73 m^2^ and discontinuing it if patients develop a sustained eGFR of the same magnitude, as it is ineffective and causes more adverse reactions. Dapagliflozin was shown to be well tolerated in hepatic impaired patients. Mild hepatic impairment did not affect overall exposure to dapagliflozin or its major metabolite dapagliflozin 3-O-glucuronide. However, patients with severe hepatic impairment have higher dapagliflozin systemic exposures. The benefit: the risk ratio should be individually assessed and managed [14].

### Role of dapagliflozin in HF among diabetic & non diabetic patients

In Type 2 diabetes patients, SGLT-2 inhibitors reduce the risk of hospitalization for HF, possibly through glucose independent mechanisms. Patients with New York Heart Association class II, III or IV HF and an ejection fraction of 40% or less were randomly assigned to receive dapagliflozin (at a dose of 10 mg once daily) or placebo in addition to recommended therapy in phase III, placebo-controlled trial. This study concludes that among patients with HF and a reduced ejection fraction, the risk of worsening HF or death from cardiovascular causes was lower in those who received dapagliflozin compared with those who received placebo, regardless of diabetes status [15]. In the Dapagliflozin and Prevention of Adverse outcomes in Heart Failure (DAPA-HF) study, patients with or without Type 2 diabetes mellitus saw reductions in cardiovascular death and hospitalization for HF in the dapagliflozin group. Three landmark trials, Empagliflozin, Cardiovascular Outcomes, and Mortality in Type 2 Diabetes (EMPA-REG OUTCOME), Canagliflozin Cardiovascular Assessment Study (CANVAS) and Dapagliflozin Effect on Cardiovascular Events (DECLARE-RTIM58), demonstrated the beneficial effects of SGLT-2 inhibitors in preventing macrovascular events and hospitalization for HF [16]. The DAPA-HF trial, recently concluded that dapagliflozin significantly improved the outcomes of patients with HF and reduced ejection fraction, regardless of the presence or absence of Type 2 diabetes mellitus. More research will shed light on the clinical significance of these drugs in the treatment of HF.

### When to use dapagliflozin as monotherapy or as a combination with other antihyperglycemic agents

Dapagliflozin is effective in lowering HbA1C in Type 2 diabetes mellitus patients as monotherapy and in combination with other antihyperglycemic agents. Its use is associated with minor weight and blood pressure reductions [5]. Dapagliflozin is effective as a monotherapy or as an adjunct to insulin or with other oral antidiabetic agent in lowering body weight and blood pressure. Dapagliflozin has been shown in clinical trials to be effective and safe in patients with Type 2 diabetes mellitus, both as monotherapy and in combination with other glucose lowering agents [17]. Therefore, when diet and exercise alone are insufficient to achieve adequate glycemic control in people with Type 2 diabetes mellitus, dapagliflozin once daily is approved for use as a monotherapy (in patients who are intolerant of metformin) and as an add-on combination therapy (with other glucose lowering agents, including insulin). Dapagliflozin as monotherapy is much benefited among people with atherosclerotic cardiovascular disease or among patient with numerous cardiovascular disease risk factors.

### Dose-dependent indication of dapagliflozin

In adults with Type 2 diabetes mellitus and established cardiovascular disease or multiple cardiovascular risk factors, dapagliflozin is indicated as an adjunct to diet and exercise to improve glycemic control and reduce the risk of hospitalization for HF and also recommended for the risk of cardiac death and hospitalization for HF in adults with reduced ejection fraction (NewYork Heart Association class II–IV). To improve glycemic control, a starting dose of 5 mg once daily in the morning is recommended. Increase the dose to 10 mg once daily in patients who tolerate 5 mg but require more glycemic control. The recommended dose for adults with Type 2 diabetes mellitus and established cardiovascular disease or multiple cardiovascular risk factors is 10 mg once daily to reduce the risk of hospitalization for HF.

### Concern on adverse effects of dapagliflozin

Urinary tract infection and genital infection have been the most widely mentioned adverse effects of dapagliflozin. SGLT-2 inhibition induces an increase in glycosuria leading to fungal infection in the perineum and genitourinary tract, frequent urination and electrolyte imbalances. Other side effects include hypotension, hypoglycemia and lower serum uric acid levels. On its own, dapagliflozin is likely to produce hypoglycemia. The ability to use new medicine combinations without developing hypoglycemia is a major strength of dapagliflozin. Glycosuria induced chronic osmotic diuresis would be the reason for hypotension. Regarding malignancy, dapagliflozin is associated with an increased risk of breast and bladder cancer [18]. The drug dapagliflozin has been proven to raise low-density lipoprotein cholesterol, hematocrit and serum phosphorus levels. Monitoring of these parameters is necessary before starting dapagliflozin dosages since increases in these laboratory parameters can have unfavorable implications [5].

### Can dapagliflozin be an option for an elderly patient?

Hospitalization for HF, cardiovascular disease, myocardial infarction rates is higher in elderly patients. While selecting the appropriate medication for elderly patients, the complexity of treatment, side effects and drug interactions are all crucial factors to be considered. Some authorities have not suggested starting dapagliflozin in elderly patients because of limited therapeutic evidence. In older individuals, few negative effects are particularly concerning. With advancing age, the risk of volume depletion increases and there is more concern regarding the potential negative implications of volume depletion in elderly individuals such as falls and renal injury. In this regard, the reported reduction in hospitalizations for HF and renal benefit with dapagliflozin is comforting, with no increased risk of volume depletion and fewer acute kidney injury occurrences [19].

### Can dapagliflozin given to overweight women after gestational diabetes?

When dapagliflozin was administered alone there was significant increase in high density lipoprotein and decrease in triglyceride concentration, glucose excursion was improved after an oral glucose tolerance test. Both dapagliflozin and metformin combination had a positive effect on body weight, waist circumference in obese or overweight women with gestational diabetes. The risk of cardiovascular complication was also reduced [20].

### Drug–drug interaction risk from five databases

Dapagliflozin, an SGLT-2 has been an effective option in treating diabetes mellitus and is also recommended for the risk of cardiovascular death and hospitalization with reduced ejection fraction. As the dapagliflozin is used as add on therapy in the existing condition, the clinical approach for the use of dapagliflozin should have major concern on drug–drug interaction and their potential interaction effects.

In this view point, we consulted five databases:

- Micromedex drug interaction (www.micromedexsolutions.com/micromedex2/librarian/PFDefaultActionId/pf.LoginAction)
- Medicine complete.com (https://about.medicinescomplete.com/)
- Epocrates (www.epocrates.com/)
- Medscape (www.medscape.com/)
- Drugs.com. (www.drugs.com/drug_interactions.html)

Potential interactions were assessed between dapagliflozin and primarily used drugs for managing comorbid conditions such as diabetes mellitus, hypertension and cardiovascular illness. The interactions resulted from consulted databases are summarized in (Table 1). The results suggest that dapagliflozin when concomitantly used with other antidiabetic agents such as metformin, acarbose, linagliptin and sitagliptin showed moderate interaction with the risk of hypoglycemia. The drug glimepiride and glipizide showed major interaction effects from more than three databases emphasizing the risk of hypoglycemia by pharmacodynamic synergism. Routine monitoring of blood glucose level is recommended. Therapy modification with low dose insulin or insulin secretagogue can be recommended to avoid the risk of hypoglycemia when co-administered with dapagliflozin. Risk of hypotension, renal impairment is the possible interaction effects between dapagliflozin and primarily used antihypertensive drugs such, as enalapril, lisinopril, olmesartan, telmisartan, atenolol, propranolol and amlodipine. The severity among these drugs is found to be moderate; however, monitoring of blood pressure and renal function test is recommended if coadministration is required. The interaction effects with enalapril, lisinopril, atenolol and propranolol are conveyed from more than three databases and so more concern is required when these drugs are coadministered with dapagliflozin. Renal impairment are possible interaction effects that may arise when dapagliflozin is concomitantly used with enalapril, lisinopril, olmesartan, telmisartan. No evidence of interaction effects is seen with concomitant use of dapagliflozin with drugs acting on the cardiovascular system. These interaction effects may or may not occur in all patients, it significantly depends on the pharmacodynamics characteristics of a particular patient. However, always monitoring of symptoms of interaction effects and adverse effects are recommended during the course of therapy.

**Table 1.** Drug interactions between dapagliflozin and drugs used for comorbid conditions.

Antidiabetic drugs	Metformin	Linagliptin	Sitagliptin	Glimepiride	Glipizide	Acarbose
Dapagliflozin	▵ Yellow	▵ Yellow	▵ Yellow	♦ Red	♦ Red	▵ Yellow
Drugs acting on cardiovascular system	Aspirin	Clopidogrel	Ticagrelor	Warfarin	Rivaroxaban	Atorvastatin
Dapagliflozin	Green	Green	Green	Green	Green	Green
Antihyper-tensive drugs	Enalapril	Lisinopril	Olmesartan	Telmisartan	Propranolol	Atenolol
Dapagliflozin	♦ Yellow	♦ Yellow	▴ Yellow	▴ Yellow	♦ Yellow	♦ Yellow

▵ Drug interaction reported from single database.

▴ Drug interaction reported from three databases.

♦ Drug interaction reported from more than three databases.

Green: No interaction; Yellow: Moderate interaction which requires caution and close monitoring; Red: Major interaction which needs monitoring, dosage adjustment or alteration of drugs.

## Conclusion

Dapagliflozin offers a significant option for the therapy of a wide range of patients, regardless of the history of cardiovascular disease, due to its antihyperglycemic, cardioprotective, and marked renoprotective qualities along with favorable tolerability profile. We anticipate that subsequent studies will shed more light on the therapeutic significance of these medications in the management of cardiovascular complications.

## Future perspective

Diabetes, obesity, insulin resistance and reduced glucose tolerance are related to increased intra-myocardial lipid levels, resulting in lipotoxicity, diastolic dysfunction and cardiac electrical abnormalities. The risk of cardiovascular disease, myocardial infarction, stroke and HF risks are all higher in diabetic patients. Once the cardiovascular disease is established, the prognosis is worse in a diabetic patient when compared with a nondiabetic patient. A relatively recent class of oral antihyperglycemic agents called SGLT-2 inhibitors increases urinary glucose excretion, which lowers plasma glucose levels and promotes two major cardioprotective effects by lowering blood pressure and its diuresis action. The cardioprotective effect of SGLT-2 inhibitors imparts the prevention of HF. Dapagliflozin, canagliflozin, empagliflozin, and ertugliflozin are some of the SGLT-2 inhibitors that are currently approved and are taken orally once a day. Among them, dapagliflozin is a highly selective SGLT-2 inhibitor with demonstrated efficacy and safety in Type 2 diabetes mellitus patients. Therefore, future studies with SGLT-2 inhibitors in a wider range of patients may suggest novel pharmacological approaches for the management of diabetic patients with cardiac complications.
